# Renting legality: How FLEGT is reinforcing power relations in Indonesian furniture production networks

**DOI:** 10.1016/j.geoforum.2018.10.008

**Published:** 2018-12

**Authors:** Ahmad Maryudi, Rodd Myers

**Affiliations:** a Faculty of Forestry, Universitas Gadjah Mada, Indonesia; b Global Environmental Justice Group, School of International Development, University of East Anglia, Norwich, United Kingdom; c Senior Research Associate, Faculty of Forestry, Universitas Gadjah Mada, Indonesia; d Dala Institute, Jakarta, Indonesia

**Keywords:** Power, Access, Global production networks, FLEGT, Legality, Certification

## Abstract

Over the past few decades, transnational and supranational market-based forest governance systems have been developed to address the complex problems associated with deforestation, by improving the legality and sustainability of timber traded in global markets. This is catalysed by the increasing global production and consumption of timber products and increasing sensitivity of interest groups to how timber products are produced. A broad range of actors is involved in global production networks. This paper discusses how hierarchies and networks of power across the timber production network are encountered and negotiated. More specifically, it investigates the power constellations of wood furniture actors in Indonesia, nested within global production networks: who holds the power, how power is gained and maintained, and who wins and loses over time. Using the case of the timber legality assurance system in the context of the European Union Forest Law Enforcement Governance and Trade (FLEGT) initiative, we demonstrate that legality verification in Indonesia is both entrenching pre-existing inequitable power relations while producing new modes of elite capture. Legality verification requires new knowledge and additional costs that are sometimes beyond the capacity of certain (particularly smaller) furniture manufacturers operators. This has driven a new practice of renting out FLEGT licenses by larger producers/manufacturers to smaller ones in the country. Although the practice implies potential risks (e.g. fines), large companies in Indonesia manage risk by drawing from pre-existing patronage relations. They also appear to find the risk worthwhile, as it produces financial gain but moreover, a new form of control over the market. Meanwhile, small operators and artisanal producers that still aspire to global markets face disproportionate challenges to engage in legality and are becoming more vulnerable as a result of new legality measures.

## Highlights

• We examine the power constellations of wood furniture actors under FLEGT.
• FLEGT entrenches pre-existing inequalities and produces new modes of elite capture.
• Legality verification drives new practices of renting out FLEGT licenses by larger producers.
• Legality verification produces new opportunities for financial gain for larger firms.
• Renting legality creates a new form of control over the market for large firms.

## 1 Introduction

New policies and market-based mechanisms in the global timber market have emerged to promote the responsible and wise use of forest resources (Tacconi, 2012). These stand in contrast to the dominance, until recently, of more regulatory approaches to forest management and timber product manufacturing and trading (such as log export bans) in producer countries. In 2003, the European Union (EU) adopted the Forest Law Enforcement Governance and Trade (FLEGT) action plan, which aims reduce illegal logging by improving forest governance and promoting trade in legally produced timber (EC, 2003). Central to the initiative are bilateral Voluntary Partnership Agreements (VPAs) between the EU and countries exporting to the EU. VPAs involve the EU and partner countries agreeing to eliminate illegal timber from the partner country’s exports to the EU (in practice- exports to all destinations) (Brown, 2008, Maryudi, 2016). Every VPA entails an assurance system that can verify timber legality. If such a system fully comes into place, then compliant exporters can merit the award of a 'FLEGT license', which will be granted through a national licensing authority established in the producer country. FLEGT was followed by the European Union Timber Regulation No 995/2010 (EUTR), which entered into force on March 3, 2013. Operators in the EU, exporters to the EU, and producers under VPAs are obliged to ensure that they trade only legal timber and wood products with the EU.

Indonesia has implemented its legality assurance system, locally referred to as *Sistem Verifikasi Legalitas Kayu* (SVLK) since 2009. In 2016, Indonesia became the first VPA country to issue a FLEGT License,^1^ with which Indonesian timber products freely enter EU markets (a so-called ‘green lane’), as they automatically meet EUTR requirements (EU-Commission., 2016, Maryudi et al., 2017a). Indonesia supplies approximately a third of the EU’s tropical timber imports by value (EU-Commission, 2016). Export values have been on the decline for the past decade (Nurkomariyah et al., 2016).

Before exports, Indonesian wood furniture follows different phases of production and distribution pathways, involving numerous actors in the production network (a related perspective to the concepts of value or commodity chain - see the next section for more), e.g. sawmillers, small or large manufacturers, traders, wholesalers, and retailers (Purnomo et al., 2014). Sawmills may include a range of small to large actors responsible for milling timbers harvested from the forest into more manageable cuts of wood before further manufactured. Furniture manufacturers include large companies, small and medium-scale enterprises (SMEs) and home industries (Purnomo et al., 2014, Kaplinsky et al., 2003, Nurkomariyah et al., 2016). They are often responsible for the design of furniture (Roe, 2015), and fulfil design specifications from buyers.

Coe et al. (2008) argue that the core of a production network relates to transforming inputs into outputs and then distributing them. The input-output structures between different phases of production and distribution shape the creation and capture of value along the production network (Henderson et al., 2002, Coe et al., 2008). As Hess (2008) notes, networks are characterised by unequal distribution of power among actors. This paper investigates the power constellation of wood furniture actors in Indonesia, nested within global production networks: who holds the power, how power is gained and/ or maintained, and who wins and loses over time*.* More specifically, we seek to understand whether and how the implementation of a legality assurance system in the context of FLEGT interacts with and affects power dynamics.

Legality verification requires new knowledge and additional costs that are sometimes beyond the capacity of certain operators (see Setyowati and McDermott, 2016, Maryudi et al., 2017b). Public data from the Ministry of Environment and Forestry (2018) shows there are 1343 verified processing, manufacturing and trading firms (excluding: forest operators, registered-only but not yet verified, and unconfirmed firm types). This figure represents only a small per centage of firms in the country, a figure for which reliable data are not available, however, Ministry of Industry source stated 140,000 firms (Agro-Indonesia, 2018, Kementrian Perindustrian Republik Indonesia, 2018), which would suggest less than 1% of firms have been verified. More specifically, nearly two-thirds of them are large operations. This highlights the enormous challenges facing Indonesian small producers. Crucially, empirical evidence on 'sustainable' production of small manufacturers, and the sustainability of small-scale versus large-scale production is very scant. Of the few current scholarly works, Nurrochmat et al. (2016) discuss the likely winners and losses of the policy change of the allocation of timber sources for furniture industries in Central Java (Indonesia). Hence, investigating how different actors respond to the new requirements, and adjust their operations, enables us to investigate who holds power, how they gain and maintain power that ultimately results in the winners and losers in the production networks.

## 2 Theoretical frames: Power within global production networks (GPNs) governance

Authority and control over natural resources have broadly evolved over time. They have increasingly shifted beyond the state (Rhodes, 1997, Maryudi et al., 2018). Jessop (2011, 108) defines the new conception as “the structures and practices involved in coordinating social relations that are marked by complex, reciprocal interdependence, and meta governance refers in turn to the coordination of these structures and practices”. In talking about governance as process, structure, practice, and space, the importance of the relations among actors becomes a critical point of enquiry. ‘Governance’ is about the “spaces in which power is encountered and negotiated” (Newman, 2005, 4), which evokes both a more broad enquiry into the range of actors that can be involved, and a deeper enquiry into the hierarchies and networks of power brokering within research boundaries.

This new way of understanding governance fits well with the context of the production and distribution of a commodity for a market. Markets are not only commercial exchanges (transfers of good, property, and money), but also the practices that enable such exchanges to take place (Sayer, 2002, North, 1991). The global production networks (GPN) framework attempts to explain input-output structures involving various actors (Henderson et al., 2002). It extends from more conventional perspectives such as Global Commodity Chains and Global Value Chains, which tend to focus less on polycentric notions of power (Bair, 2009, Coe and Hess, 2006, Coe and Hess, 2013). In GPNs, a broad range of actors interact, with own interests and agendas, seeking influence to capture value. In that context, Levy (2008) argues that GPNs are not simply chains of value-adding activities; they relate how authorities are constructed within markets and their associated distribution of resources. Thus, ‘governance’ is about the power relations that result in winners and losers within the networks (Hess, 2008).

The new conceptualisation of power in GPN governance recognises power relations in a production system are complex (Coe and Hess, 2013). While large companies typically hold great influence over GPNs, other actors may also wield powerful tools (Gibbon and Ponte, 2008). For instance, Myers (2015) found that intermediaries had immense control over the supply of resources, but lacked influence on the policies that affect trade. They can also squeeze significant profits out of producers (Maryudi et al., 2015, Myers, 2015). In GPN governance thinking, power may be centred or de-centred and is subject to change (cf. Hess, 2008). This is a marked shift from conceptualisations of governance and power in predecessors to GPNs (‘value chains’ and ‘commodity networks’) in that an enquiry into governance must go beyond static assumptions of power, and rather examine how power is gained and maintained or lost.

Power is manifested in relational effects of social interaction in which actors seek mechanisms to gain and maintain access to, and control over, benefits (Coe et al., 2004, Ribot and Peluso, 2003). This conception of power is also embedded within the concept of access, which is defined as “a bundle of power” (Ribot and Peluso, 2003). Control of access and the ability to benefit from something, as Ribot and Peluso (2003) conceptualise, can respectively be equated to “power over” and “power to” in the power literature (Lukes, 2005). In GPN governance, actors may influence regulatory structures, control critical resources, and construct discursive legitimacy (Levy, 2008, Hardy and Phillips, 1998, Peppard and Rylander, 2006); this reflects the general dynamics of power (Levy, 2008). For example, product standards are often at the centre of intense struggles as they facilitate or hinder the participation of particular actors (Bolwig et al., 2010). In the context of FLEGT, large producers in some countries have lobbied governments to institute cumbersome harvest regulations which drive small-scale loggers and producers into illegality (Obidzinski et al., 2014). With regard to critical resources, contacts with and information about international buyers are usually controlled by large firms or associations that create dependencies among smaller manufacturers (Purnomo et al., 2011). FLEGT may further reshape forest discourse resulting in the changing power relations in favour of or against certain actors (Ochieng, Visseren-Hamakers, and Nketiah, 2013).

## 3 Research methods

This research followed critical social science approaches to justice complement the normative approaches of legal scholars and philosophers (eg. Rawls, 1999). Critical social science approaches do not start from a particular theoretical position, such as Rawls’ theory of justice as fairness, but instead, compare and contrast people’s actual claims about (in)justice (Walker, 2012, Sikor, 2013).

To answer our research questions, we conducted 100 in-depth interviews (Table 1) with timber manufacturers (small, medium, large manufacturers, as well as exporters and non-exporters), intermediaries, and exporters from three main furniture producing regions in Java Island: Jepara District, the Greater of Surakarta (Solo, Klaten and Sukoharjo Districts), and Special Region of Yogyakarta (Sleman, Gunungkidul, Yogyakarta and Bantul Districts), with some additional interviews in the timber producing regions of Blora and Rembang, where the occurrence of illegal cutting is high. We also interviewed 40 representatives from associations, policy-makers (at national and sub-national levels), NGOs (at national and local levels), and key informants from a variety of organisations/institutions dealing with legality verification in Indonesia that include verification bodies and independent monitoring networks.

**Table 1:** List of interviews.

No	Actor types	Number of interviewees	Notes
1	Sawmillers	10	Both registered & informal
2	Wood traders/intermediaries	15	
3	Furniture manufacturers		
	- Small & medium scale	30	20 of which are exporters
	- Large scale	15	
	- Artisans/home industries	25	
4	Chainsaw men	5	Individual illegal loggers
5	Associations	5	
6	NGOs & civil society groups	6	
7	Policy makers		
	- National	4	
	- Sub national	12	
8	Verification bodies	5	
9	Donors	3	United Kingdom-Indonesia’s Multistakoholders Forestry Programme
10	Academics	2	
11	Consultants	3	

We started our investigation by interviewing key informants, who we also asked to identify actors whom we could interview within the production network. From there, we followed these actors’ trading partners both upstream and downstream. We used our own experience and professional judgements in selecting non-industry respondents who may provide insight into power issues in furniture production networks in Indonesia (e.g. government regulators, industry associations, NGOs etc, which are not involved in trading directly, but play an important role in governance and are in a position to provide insights). The first author has been involved in FLEGT and legality policy since the formative phase. In each interview, we focussed on governance and justice issues. For market-actors (corporates, manufacturers and wood traders) we also asked about how they operate, the market conditions (e.g. sales), and changes, if any, prior and after the implementation of legality verification in Indonesia. We further employed content analysis on media, specifically on the issues of fairness and the impacts of legality verification in Indonesia. We acknowledge a limitation in our data pertaining to the ability to measure change in key indicators, like profit for example, pre and post SVLK/FLEGT, however propose that our qualitative analysis provides insight into these issues and are more useful to understand the core of our paper: which is about changes in the power relations among actors.

## 4 Global furniture markets and production networks in Indonesia

This section briefly discusses the global trade of furniture and the extent to which Indonesian furniture is nested within. It also provides a brief overview the general market conditions and the domestic production structures, which are characterised by a high number of informal small-medium scale manufacturers/home industries, and their reliance on large firms for export operations.

Global trade of furniture has grown from USD 94 billion in 2009 to USD 135 billion in 2014 (CSIL, 2016) and USD 140 billion in 2016 (UNECE, 2017). In both production and consumption, the global South is increasingly dominant (UNECE, 2017, Purnomo et al., 2009). Middle and low-income countries have assumed more than half of global furniture production since 2010. This shift is remarkable considering that in 2003, three-quarters of furniture production was in high-income countries. By 2012, almost 60 per cent of production was in middle to low-income countries, dominated by China (Renda et al., 2014). The share of furniture consumption in high-income countries has also dropped, from 82 per cent in 2003 to 53 per cent in 2012 (Renda et al., 2014). In 2016. the biggest importers in Europe were Germany, France, and the UK (UNECE, 2017).

Over the past twenty years, Indonesia has been one of the major exporters of furniture but its position in the global trade has dropped significantly (Nurkomariyah, Firdaus, and Nurrochmat, 2016). In 2015, with trade values of USD 1.81 billion, it ranked only the 25th, dropping from the 5th in 2000 (Salim and Munadi, 2017). Respondents reported closures of some manufacturing firms over the past few years. Recent estimates show that there are more than 100,000 primary and downstream processing industries; 95 per cent of them are SMEs (Purnomo et al., 2014). Most timber processing industries are labour-intensive and tend to operate in clusters (Melati, Purnomo, and Shantiko, 2013). The National Statistics Bureau estimates that small-scale wood and handicraft enterprises employ up to 1.5 million people (BPS, 2015). Further, a large proportion of the companies are unregistered (i.e. informal). For instance, in the Special Region of Yogyakarta, unregistered manufacturers make up approximately 75% of the total furniture enterprises (Obidzinski et al., 2014).

Indonesian small-scale furniture manufacturing industries supply both domestic and international markets (Andadari, 2008). Most furniture companies are export-oriented. Domestic markets are estimated to only absorb 6–10% of the production although several manufacturers started to target them over the past few years (Salim and Munadi, 2017). For domestic markets, they supply a network of furniture shops across the country. Marketing is based on contacts between networks of small firms and specific traders that are linked to specific furniture shops (Burger and Smit, 2001, Purnomo et al., 2014, Purnomo et al., 2009). Internationally, they act as subcontractors and are involved in production networks managed by large firms and traders (Kusmantini, Guritno, and Rustamaji, 2015) due to lack of marketing skills and information (Purnomo et al., 2014, Van Geenhuizen and Indarti, 2005, Maryudi et al., 2015, Maryudi et al., 2017a, Maryudi et al., 2017b, Maryudi et al., 2017c). Subcontracting allows small firms to concentrate on production and leave the management and the risk of the market, with its changing tastes and fashions, to the lead firms and merchants (Burger and Smit, 2001). In most cases, production, models, quality, and other requirements are pre-determined by the buyers (Andadari, 2008), which are often subsidiaries of overseas retailers (Purnomo et al., 2014).

With regard to the exports, a small number of large firms and traders make contracts with global buyers, wholesalers or retailers. They play an important role as a trade hub, and therefore have high bargaining position *vis-à-vis* small manufacturers (Purnomo et al., 2014). In the past, as Kato (2005) argues, they exercised power and gained strong influence in the production-distribution networks through steering the exports registration body *Badan Revitalisasi Industri Kayu* (BRIK, Indonesian Institute for the Revitalization of the Timber Industry).^2^ As registered exporters (*Eksportir Terdaftar Produk Industri Kehutanan*, ETPIK), they controlled timber exports and brokered local manufacturers with international buyers. With the introduction of SVLK, ETPIK registration is no longer in place. Exports can now be conducted by any manufacturer or trader with either a FLEGT License or a V-Legal depending on the destination (V-Legal - Verified Legal - is for non-EU destinations). Under the current regulation, non-manufacturers remain eligible for legality verification and conducting exports, so long as the products are supplied by legally-verified manufacturers.

## 5 Production network governance in the era of legality requirements

The first part of this section briefly discusses Indonesia’s exports following the implementation of legality verification. It reveals how national level data is insufficient in capturing dynamics in production-distribution networks and changes in power relationships and business operations. The ensuing two parts analyse the different experience by small manufacturers and large firms based on our interviews- how they respond to the legality policy - and reveal the power constellation of wood furniture actors in Indonesia, who wins and who loses. In these parts, we show that the renting of certificates has become evident following the imposition of new legality complexities, the reasons why small manufacturers rent, and then why big ones rent out, and how these reinforce the pre-existing patronage relations.

### 5.1 Indonesia’s exports following legality verification

Access to, and integration of, Indonesian timber products in global markets have proven to be two of the thorniest issues since the Government of Indonesia commenced FLEGT VPA preparation (formulating a legality policy) and subsequent negotiations with the EU. The formal interests of the central forest bureaucracy relate to broad goals of improving domestic forest governance through eradicating illicit forest activities (ID-108). During the VPA negotiations, the Government of Indonesia also articulated its interests in enhanced sales in European markets through the eventual agreement (Maryudi, 2016). Concerns about the adverse impacts of legality verification raised by small forest operators and artisanal timber manufacturers emerged as a major concern during the formative phase of the national legality system (Setyowati and McDermott, 2016, Lomax, 2014, Nurrochmat et al., 2016). Although this was later responded to with streamlined standards for small operations, the concerns did not come to end.

Following the full implementation of the legality system, the forest authority regularly provides updates on exports of forest products using V-Legal licenses, in terms of volume and values, indicating that both have increased. A high-ranking official with the Ministry of Environment and Forestry said that the exports’ values in 2015 increased by nearly 50 per cent of the previous year, from US$6.6 billion to US$9.9 billion (BisnisIndonesia, 2016). The export values also increased from 15.73 million tonnes in 2015 to 17.46 million tonnes in the following year (Endarwati, 2017). Focusing on the national aggregates, in terms of both metrics and values, ignores key political dynamics within the production networks at the national and local levels. While the production networks are quite complex and characterised by power asymmetries among actors, as previously discussed, the data are not sufficient to reveal who within the networks enjoy the most benefits and who become disadvantaged. As an online database of the Ministry of Environment and Forestry (2018) shows, legally-verified processing, manufacturing and trading entities are dominated by large firms (processing over 6000 m^3^ per year) representing 858 (63.9%) of 1344 firms. In contrast, only four (0.3%) of verified firms are home industries.

In our interviews, informants from the government bureaucracies, verification bodies and donor agencies (ID-114, 027, 104, 106, 108) remain enthusiastic about the improved integration of small manufacturers in global markets. They indicated that the implementation of legality verification reduces the reliance of small firms and manufacturers on large traders/ exporters by linking them directly with international buyers. They further claimed that international buyers actively sought information on legally-verified manufacturers regardless of the size of their operations (ID-106). However, political uproars and strong resistance from the national association of small furniture and handicraft manufacturers (*Asosiasi Mebel dan Kerajinan Indonesia*/AMKRI) cast a shadow over the enthusiasm of the state and large companies. The association claims that legality verification puts more financial burdens on small operations and impedes their integration in the global markets (see Purukan, 2015). This partly explains the several delays for the full implementation of the legality policy during which SMEs were only required to make a declaration (*Deklarasi Ekspor*/DE) that the exported products originated from legal sources rather than meet the full eligibility requirements for SVLK.

Our data show that different GPN actors experience timber legality in different ways. Regarding changes in sales volumes and prices, our respondents, both domestic and export-oriented manufacturers/ traders, suggested both a decline (ID-125, 126, 144) and an increase (ID-16, 17, 19, 22, 23, 25, 40, 145). However, a number of SMEs are reported to have experienced hard times and were facing much stiffer competition in the global markets from cheaper products in other exporting countries, which do not impose a similar legality policy (see Anon, 2015). Therefore, statistical improvements in export volumes or sales hardly reflect how individual companies have been affected by timber legality laws. We therefore concentrate on exploring some of these differential experiences in our empirical discussions below.

### 5.2 Small manufacturers and artisans: Renting legality certificates as a survival strategy

Legality requirements increase expenditures associated with changes in operations (e.g. the elaboration of more detailed inventory documentation and employee training) as well as actual verification and surveillance costs. Although the legality requirements for small operators have been made much simpler than the standards for the larger and more integrated operators (Maryudi et al., 2015), numerous respondents described the burdensome registration procedures,^3^ in terms of time and financial investment, as a primary reason behind their reluctance to pursue legality verification. Recent efforts to simplify registration procedures^4^ do not allay fears among informal manufacturers (MFP, 2015). Small manufacturers, particularly home industries, remain constrained by the poor documentation and administration of raw materials, timber balance (input and output), products and orders, all of which are crucial for legality verification. While some of these actors may turn their attention to domestic markets (which still requires proof of timber legality, but is subject to less scrutiny), many of them derive significant sales through sub-contracts from exporting companies and therefore engage in ‘renting legality’. A company representative (ID-124) explained that they would need to employ at least two trained workers to process administrative requirements, whose salaries might compromise profits. Formal safety and health standards are also seen as too costly (see Yovi and Nurrochmat, 2018). Finally, exposure to the taxation office is perceived as a significant threat to profit margins (Maryudi et al., 2014). By keeping their business unregistered, small manufacturers can avoid paying taxes for any transactions (e.g. ID-91).

In order to meet legality verification requirements, some small manufacturers we interviewed (ID-20, 40, 41, 46) rely on expensive ‘legality consultants’, who offer their services to ensure their clients confirm to legality requirements. These consultants, charge IDR 100–150 million (USD 7300–11,000) depending on the size of the business.^5^ Even when the legality certificate is secured, it must be maintained through monitoring, which costs an added IDR 25–30 million (USD 2000–2500) every other year. We found frequent reference to small furniture business closures, citing the implementation of legality policy as one of the main driving factors (see Anon, 2015). Our research also found that several actively exporting manufacturers (ID-09, 11), simply quit the timber business. An exporter (ID-11) gave some insight into the broad implications of legality verification:

*“timber business is now becoming unbearable with so many requirements that keep changing and increasing. The legality verification is too much for us. Not only the financial consequences, it obliges us to change our day to day practices, it’s complicated and indeed costly. At the same time, we don’t see any significant benefits by engaging in legality. Better for us to completely quit the business. I observe more promising business with less complicated procedures and requirements.”*

In response to the multiple burdens presented by legality requirements especially on small manufacturers, the practice of renting V-Legal and/ or FLEGT-License legality certificates has arisen. This appears to be a new iteration of past efforts to bypass legality procedures: under the aforementioned BRIK and ETPIK in which export certificates were sold illegally on the black market (see Colchester et al., 2006, Gellert, 2010, Myers, 2015). Certificate renting was confirmed by several manufacturers on both the renting out and receiving ends (ID-117, 121, 122, 124, 126). Civil society groups (ID 107, 109), verification bodies (ID-106) and donors (ID-108, 123) also acknowledged this practice. In an attempt to downplay the magnitude of the practice, a respondent from the Ministry of Environment and Forestry (ID-92) argued that the legality verification may take time to properly function.

Certificate renting occurs for a number of reasons. The primary rationales we found in interviews were (1) bureaucratic avoidance, and (2) limited access to financing. We explore these issues now.

First, as previously mentioned, legality verification involves additional technical and managerial complexities. A small manufacturer (ID-122) suggested that he is forced to follow the practice because “*exports are complicated. [I] do not have the knowledge. [I] do not know the tax regulations. [It is] much simpler relying on larger companies as they will deal with the required processes including how to deal with the customs”.* Another company representative (ID-126) added the exporting company will look after all of the necessary documents for his exports; he just provides data such as the products, specification, and volume.

Second, limited access to financing encourages SMEs to borrow the legality certificate for the exports of their products. This is a main driver for many small manufacturers. According to respondents, managers and owners behave rationally, exercising the best possible return from their business. Small manufacturers and artisans with less frequent exports see renting legality certificate as the much cheaper option despite the fact that it also pushes their profits down. One small-scale manufacturer (ID-124) gave detailed insights:

*“Before having SVLK, we used the certificate owned by other companies. We just needed to pay them. In addition, there are some non-producer companies that provide services for non-certified industries. We pay IDR 4.5 million IDR / container. Many small industries in [this region] use such service as they do not have their own SVLK. I am unsure whether or not the practice is forbidden, but there are many (small) industries that use that. For small industries, they prefer [renting] the legality certificate as it is much cheaper. Assuming they only export 5-10 times a year, [renting] a certificate is much cheaper than an SVLK which could cost them 50 million IDR. In addition, they do not need to employ additional workers to deal with SVLK. For doing that, they need two employees, who are paid in total 48 million IDR per year. Requirements for SVLK are complex, e.g. for documentation. Small industries are not used to that. So, there are two main reasons why small industries do not apply SVLK: the costs and the complicated procedures and administration, which also costs a lot. On top of that, they do not need to worry about taxes. When we use the certificate of other companies, we need to provide photos of products that will be shipped and the packing lists, the products and their volume”*

### 5.3 Large companies and exporters: Renting legality licences for accumulating more financial benefits and enhancing control in the markets

Small producers saw using larger exporter’s FLEGT or V-Legal certificates as a strategy for survival in the face of the financial burdens and administrative complexities of legality verification and new trading regulations. By so doing, however, they make themselves more vulnerable. Large firms and exporters renting their licenses are perfectly positioned to capture more benefits and consolidate their control and power in the GPN. During the BRIK era, exporters of timber products were distinguished into: producing and non-producing ETPIK holders; both collected timber products from small firms and artisans and acted as a ‘forwarder’ or intermediary to global markets. Although ETPIK is no longer required for exports, legality regulations still allow pure intermediaries (non-producers) to apply for legality verification and thus conduct exports under the requirement that they establish contracts with legally-verified producers.

We identified several mechanisms by which large exporting firms, both intermediaries and producers, rented their legality certificate and continued to act as powerful actors in the GPN networks. Several respondents suggested that many of the current legally-verified intermediaries were the large exporters holding non-producing ETPIK holders under the previous BRIK system. They either conduct minor finishing activities to be categorised as a producer (ID-123) or are fictitious manufacturing firms that barely have production activities (ID-109) to absorb products from non-legal suppliers. As previously said, non-manufacturers are only eligible for legality verification with supplies from legally verified sources. Manufacturing exporters mix their legally verified and non-legally verified products supplied by small operations. One key informant (ID-123) detailed the technical complexities for auditors to check products when a certain exporter has numerous suppliers, which may even operate in distant regions. Audits may not reach the minimum number required for checking because the verification fees are often set low in the lights of competition among verification bodies. Aside from (illegally) renting certificates to small producers, large firms may deal with buyers who directly purchase products from legally-verified producers (ID-121). Other firms also exported more products by using the legal certificate of other producers. This practice is to avoid more substantive investments in a new registration as a larger operation and a new verification with a different set and more robust standards.

There are risks, as renting certificates violates regulations. One respondent from a verification body insisted that renting certificates cannot be tolerated; he suggested that there have been cases of both suspension and withdrawals of legality licenses. As previously mentioned however, relaxed verification procedures, particularly by those with a large number of clients, still opens opportunities for exporters to continue the practices in the hopes of substantive profits and other benefits relating to controls in the GPN (ID-123). One respondent (ID-109) suggested that these companies earn money from “the business of legal certificates” by renting the documents for exports to small firms. Several small manufacturers we interviewed (ID -121, 122, 124, 125, 126) highlighted that by renting legality licenses, large firms squeeze IDR 4–8 million (USD 300–600) per container. This figure makes up approximately 5–10% of the export value, depending on the products.

More importantly, certificate holding companies require sensitive business information from the smaller companies using their certificates in order to comply with SVLK. They therefore gain information on the sale prices and client details. Some smaller manufacturers suggested that under SVLK, they were disconnected from their international buyers after renting legality documents from other exporters since the market transaction is now through an intermediary rather than directly as before SVLK. This gives rise for the potential of large business to at very least outprice their smaller competitors, or attempt to woo the clients into buying from them. Although they were unsure of the exact driver, export intermediaries often mentioned either the low quality of their products or “recessions in international markets” so that the buyers had made no more purchases. One certificate borrower (ID-91) provided more candid insights:

*“I have been doing exports of furniture for about twenty years, experiencing ups and downs in the business. I have been borrowing legality certificate from larger firms to conduct exports. Using their certificates, I was able to export 10-12 containers a year to Germany, France, Italy, and the Netherlands. It lasted only three years. Since 2014, I have lost all of my European buyers. It is a high possibility that my European buyers are now served by the certificate lenders. While I still borrow legality certificate from another large firm, now I can only export 2-3 containers a year to Asian buyers. Even so, I am unsure if I can maintain this export activity”*

While getting responses from large firms was extremely difficult in our study, one key informant from the Multistakeholder Forestry Programme (ID-124) also added that the certificate owners are effectively “hijacking information on the buyers”. He provided examples in which larger companies stole the knowledge on the buyers and trading system. Several of them took advantage of this new-found access to market relations during the transitional phase of the declaration of exports that was intended for small manufacturers. An investigation report by the Indonesian Independent Forest Monitoring Network (JPIK 2016) reveals that they used names of SMEs and even falsified the declaration documents to confirm V-Legal certification. Another respondent (ID-109) concluded that the certificate lenders are the winners in the networks.

“W*hen purchasing products from small manufacturers, the payment is made after they got the money from the buyers. So basically they need zero capital, only the ability or knowledge on the markets, and the ability to violate SVLK regulations. They are big because they know the markets networks.“*

## 6 Discussion

Producing, distributing and integrating timber products in the global economy involves a broad range of actors. The way they influence the networks and compete for the capture of the values created in the networks represent power relations (Coe and Hess, 2013). In this paper, we questioned whether or not the implementation of timber legality verification, framed under FLEGT initiative, changed the power constellation of wood furniture actors in Indonesia, nested within global production networks; and how the actors behave and respond to the changes with regard to their respective business operations.

FLEGT has an objective on stimulating good forest governance including the good intention to reform the forest power constellation. We found that legality verification perpetuates the hegemony of large manufacturers and exporters by utilising their know-how capacities and capitalising on the inability of their smaller competitors to engage in legality verification. In other VPA partners, such as Cameroon and Ghana, FLEGT is also perceived to result in further expansion and power concentration of the already dominant large industries (Carodenuto and Cerutti, 2014, Wiersum and Elands, 2013). In a different context, smallholder timber producers in Indonesia are also said to be at to be disadvantaged by the implementation of legality policy (Maryudi et al., 2017b, Nurrochmat et al., 2016, Maryudi et al., 2017c).

Over the past few decades, large firms continued to reinforce their power in creating, enhancing and capturing value in global production networks (Dauvergne and Lister, 2010). Our study found that larger-scale actors possess more competences in terms of accumulating knowledge (see also Malerba, 2002), and controlling strategic resources (see also Hardy and Phillips, 1998, Ribot and Peluso, 2003). In our case, this was evidenced in meeting the legality requirements for exports under the FLEGT regime. The unequal distribution of power is exacerbated by the way small manufacturers find strategic advantages or are forced to partner with their larger competitors. This change in network structure furthers the disintegration of smaller producers from the global economy.

There might be arguments that legality verification policy in Indonesia could be accessible for all types of actors after the implementation of a more streamlined set of standards for small operations. Our research reveals that to engage in legality verification, small industries are constrained with technical, administrative, organisational, knowledge and financial barriers that further weaken their bargaining positions in the global networks. There is a growing understanding that the number of disadvantaged small manufacturers is significant, as so far only a small fraction of furniture manufacturers have been legally verified. Although detailed statistics are unavailable due the lack of registration of many of these business (the number of companies registered for legality verification is known, but the total number of companies is not known, and due to the complicated nature of contract and home-industry arrangements, numbers of companies are not representative of the total number of actors engaged in the sector).

More importantly, our research revealed how companies reacted to their limitations to participate in the timber and wood product business. Several operators resigned in a signal of acceptance that legality verification surpassed their capacities, eventually switching their focus to domestic markets or even closing down their businesses. We identified a number of small firms that adapted the legality policy by renting legality licenses to stay connected with the global economy, albeit at arm’s length from buyers. These actors saw borrowing legality certificates as a strategy of survival to deal with the administrative hassles of legality verification, trading regulations, and the associated costs.

In contrast, larger firms have organisational and financial strengths to engage in legality verification directly. We found that possessing legality certificates further enhanced their bargaining positions in the production networks. They captured more value in the GPN by brokering legality by lending their certificate to small suppliers and squeezing profits out of them. Coe et al. (2004) argue that “value can take the form of technological rents by way of access to particular product or process technologies”. There are indeed risks and potential sanctions of such practices, notably the suspension or cancellation of their certificates. Many large firms utilised loopholes in the legality policy to mask their illegal operations, principally through the ‘contractual mechanisms’ with small manufacturers in which they subsume timber for which legality is unclear into inventory that is verified as legal (see Acheampong and Maryudi, forthcoming for more strategies to use loopholes in policy).

## 7 Conclusions and policy recommendations

Our findings have far-reaching implications that are under-recognised. VPAs are implemented to provide a range of benefits, including economic opportunities to local timber enterprises (Carodenuto and Cerutti, 2014). We make it clear that FLEGT further entrenches power constellations in the production networks as large operations continue to enjoy more benefits and control (Eba’a Atyi et al., 2013). We have shown that more powerful traders are able to extract additional fees from smaller actors and gain access to valuable client and pricing data in the process. While differential powers among actors would exist without FLEGT (and indeed did before) we suggest that there are several ways in which these imbalances are exacerbated due to the FLEGT VPAs. This reinforces the argument that FLEGT VPAs are yet another forestry sector intervention that, problematically, relies on market-based mechanisms to resolve problems in large part created by those same mechanisms (Rutt et al., forthcoming). In addition, actors envision FLEGT and SVLK as policy instruments to improve forest governance, e.g. eliminating illegal practices, corruption, and to provide credible assurance to end consumers about how the timber products they use originating from responsible (legal) practices. Without addressing the possible positive changes to forest governance as a result of SVLK and FLEGT, which are outside the scope of this paper, our findings on renting legality documents illuminate challenges to improve the credibility of the system. Earlier, FLEGT and legality verification was hypothesised as providing a foothold for sustainability by improving the domestic forest governance (Benjamin Cashore and Stone, 2012). It is also praised for improving benefits for the private sectors in EU’s VPA partner countries (Carodenuto and Cerutti, 2014).

Our analysis does not disregard the potential of FLEGT to enhance timber product governance and possibly even reduce illegal logging. However, we provide insights for policy-makers to improve governance arrangements to ensure that new accountability systems do not gain legality traceability at the cost of incentivising corruption and further concentrating power within the GPN actors. The central policy issue that SVLK is designed in such a way that it provides opportunities for the larger companies to become more powerful and presents challenges for smaller actors. There has been considerable debate in Indonesia in the design and implementation of SVLK, especially in relation to the burden that it places on smaller actors, and our findings support exploring further options to develop less financially and administratively burdensome processes for these actors so they can remain competitive without being compelled to engage in the loopholes we have framed as ‘renting legality’. Devising a pro-small actor timber legality verification system would entail simplifying processes, providing increased capacity building to navigate the simplified processes, and reducing the cost burden for engaging in verification processes.

## Footnotes

1 Exports to other countries than the EU members use V-Legal documents. In this paper, FLEGT License and V-Legal, and legality requirements, are used interchangeably.

2 This body was formally charged with charged with monitoring and verifying of legal timber(Maryudi, 2015; Cashore et al., 2016), but ensured the interests of its own members by setting permits and quotas of exports (Kato, 2005; Luttrell et al., 2011).

3 SMEs are required to obtain the following documents: Building Construction Permit (*Ijin Mendirikan Bangunan*/IMB), Deed of establishment, Trading Business Permit (*Surat Ijin Usaha Perdagangan*/SIUP) or Industrial Registration Number (*Tanda Daftar Industri*/TDI), Environmental disturbance permit (HO permit), Company Registration Number (*Tanda Daftar Perusahaan*/TDP), Taxpayer Identification Number (*Nomor Pokok Wajib Pajak*/NPWP), Environmental Impact Analysis and Monitoring Activities, Industrial Raw Material Fulfilment Planning (*Rencana Pemenuhan Bahan Baku Industri*/RPBBI). Application of those documents are sequential in nature. For example, IMB is required for their HO application.

4 Several district governments started to restructure their bureaucracy through integrated one-stop services (Layanan Terpadu Satu Atap) in part to expedite SVLK implementation.

5 This figure includes the costs of actual legality verification.
